# Successful Retrieval of Rota Burr After Driveshaft Fracture

**DOI:** 10.1155/2024/5482922

**Published:** 2024-06-17

**Authors:** Tsuyoshi Kobayashi, Takeo Horikoshi, Toru Yoshizaki, Akira Sato

**Affiliations:** Department of Cardiology University of Yamanashi, 1110, Shimokato, Chuo, Yamanashi 409-3898, Japan

## Abstract

Rotational atherectomy is an effective procedure for heavily calcified lesions and those that cannot be crossed using conventional percutaneous coronary intervention (PCI) devices. Here, we report a rare case of intracoronary burr entrapment in the coronary artery due to burr disconnection from the driveshaft. A 67-year-old man undergoing hemodialysis for nephrosclerosis presented with exertional chest discomfort. Coronary angiography revealed stenotic lesions in the right coronary artery, and PCI was performed using a Rotawire Floppy. During the procedure, the disconnected burr was successfully removed without surgery using the child-in-mother technique with a guide extension catheter. Notably, the patient remained hemodynamically stable throughout the procedure and his recovery was uncomplicated. He was discharged on the second postprocedural day. At the 6-month follow-up, the patient remained asymptomatic with no evidence of myocardial ischemia. This report informs clinicians of the possibility of burr disconnection and the non-surgical intervention used for its removal.

## 1. Introduction

Percutaneous coronary intervention (PCI) for heavily calcified coronary lesions remains a challenge in the contemporary drug-eluting stent era [1]. Rotational atherectomy is useful; however, its use can lead to complications including coronary artery damage, slow flow, transection of the Rotawire, and burr entrapment, which can be fatal [2]. In this report, we present a case with a rare complication of Rota burr disconnection from the driveshaft within the coronary artery.

## 2. Case Presentation

**Figure 1:**
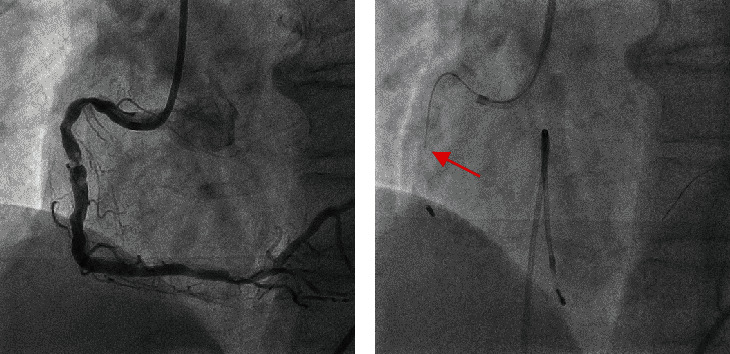
(a) Baseline coronary angiogram. (b) The driveshaft is separated from the burr (the red arrow indicates the edge of the driveshaft).

**Figure 2:**
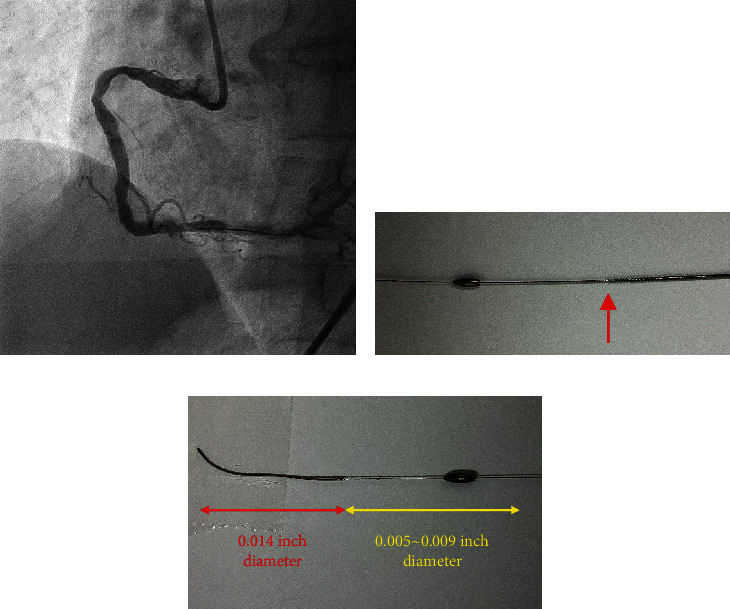
(a) Final angiogram. (b) Separated burr and edge of the driveshaft (red arrow). (c) The distal tip and body of Rotawire Floppy.

In this study, we report the case of a 67-year-old man on hemodialysis for nephrosclerosis who developed chest discomfort on exertion. Myocardial perfusion scintigraphy revealed myocardial ischemia from the inferior to the lateral wall. Coronary angiography revealed a bent moderately stenotic calcified lesion in the proximal right coronary artery (RCA), along with a severely stenotic lesion in the middle section of the RCA with severe calcification (Figure 1(a)). For PCI, a 7-Fr Judkins right (JR4.0) guide catheter was engaged through the femoral artery, and a SION blue wire (Asahi Intecc, Japan) was crossed into the distal RCA. As intravascular ultrasound (IVUS) and small-diameter balloons did not pass (Video 1), a microcatheter was used to replace it with a Rotawire Floppy (Boston Scientific, Marlborough, MA, USA). The 1.5 mm burr was advanced by cutting the proximal part of the RCA at 180,000 rpm with a slow pecking to-and-fro motion with runs of up to 20 s. The rotational speed was maintained above 175,000 rpm. Subsequently, the severe stenotic lesion in the middle section was ablated. However, we observed that the burr detached from the driveshaft as it advanced into the distal RCA despite the driveshaft being pulled (Figures 1(b) and 2(b), Videos 2 and 3). Nonetheless, the coronary blood flow was preserved, and the Rotawire was not torn.

**Figure 3:**
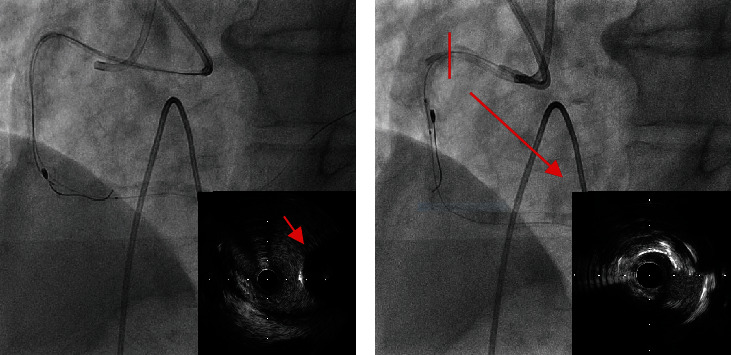
(a) The burr was observed using intravascular ultrasound (IVUS). The burr was not entrapped at this site. (b) Inflated 2.5 mm balloon next to the entrapped burr. The inner side of the proximal lesion of the bending RCA was observed by IVUS to be ablated.

A second guidewire was advanced from the second guide catheter (7-Fr JR4.0) to the distal RCA, and the burr was observed using IVUS. Notably, the burr was not entrapped at this site (Figure 3(a)). The Rotawire Floppy tip was then used as an anchor to pull the burr toward the proximal RCA, facilitated by the 0.014-inch diameter of the Rotawire tip. However, the burr was entrapped within the calcification of the mid-RCA stenosis.

**Figure 4:**
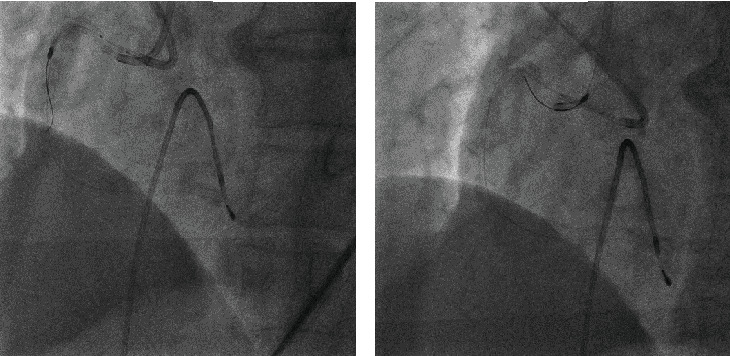
(a) Advancement of GUIDEPLUS until contact with the burr. GUIDEPLUS and burr were then pulled together. (b) Successful extraction of the burr into the guide catheter.

In response to the burr entrapment, we cut the driveshaft sheath, driveshaft, and Rotawire near the advancer and pulled the driveshaft sheath out. Attempts to advance a snare to the burr proved unsuccessful. We used the Rotawire to advance a 6Fr guide extension catheter (GUIDEPLUS; NIPRO, Japan) toward the proximal portion of the burr. However, it failed to pass through the proximal stenosis. Therefore, we dilate the proximal part of the RCA using a 2.5 mm balloon (NEON NC PRO 2.5/10 mm, 20 atm; Cordis, FL, USA) through the second guide catheter, followed by dilation at the site of the burr (Figure 3(b)). We then advanced the guide extension catheter until it was in contact with the burr and pulled them together (Figure 4(a)), successfully extracting the burr (Figure 4(b)). Finally, a Xience Skypoint 4.0/15 mm (Abbott Laboratories, Chicago, IL, USA) was implanted in the mid-RCA and the proximal part was dilated with a drug-coated balloon (SeQuent Please 3.5/20 mm; B BRAUN, Frankfurt, Germany) to complete the treatment (Figure 2(a)). It took about 80 minutes to extract the burr from the fracture. The patient remained hemodynamically stable throughout the procedure, and his recovery was uncomplicated. He was discharged on the second postprocedural day. Follow-up at 6 months indicated that the patient remained asymptomatic with no evidence of myocardial ischemia.

## 3. Discussion

Cases of burr detachment from the driveshaft are considerably rare [3, 4], highlighting the importance of reporting the bailout technique employed in such cases. Although the cause of burr tip disconnection, in this case, was not determined, it can potentially occur during burr activation when trapped or when the burr is pushed despite resistance within the stented or bent segment [4]. In the present case, the Rotawire floppy was used to prevent the strong guidewire bias. However, IVUS revealed ablation on the inner side of the proximal lesion of the bending RCA (Figure 3(b)). We set the platform at 1~2 cm proximal from the bend. The bend in the driveshaft substantially increases the friction force between the lesion and the driveshaft. This torque may have persisted at this site, potentially leading to entrapment due to the force applied to the periphery when cutting the middle lesion of the RCA. We have identified two areas for improvement. The first is the guiding catheter. Multiple passes for angulated lesions to improve passage resulted in deep cuts, which may develop into perforations. We used a JR guide catheter which was pushed back during rotational atherectomy. In an unstable system, operators must excessively push the burr, which is closely associated with “jump in.” The second is the location of the platform. Despite the scarcity of studies on how to move the platform during the procedure, it is suggested that setting the platform distal to the bend and ablating the mid-RCA calcification can reduce friction between the driveshaft and the lesion at the bend, potentially preventing driveshaft fracture.

In the present case, the disconnected burr was pulled proximally, using the Rotawire tip as an anchor with a 0.014-inch tip (Figure 2(c)). When the burr separates, lesion preparation on the proximal side is insufficient, and Dynaglide rotation is not feasible; therefore, the burr is likely to be trapped prior to retrieval. In the case of burr entrapment, several percutaneous bailout methods have been reported [5, 6]. These methods include manually pulling back the Rotawire, repeatedly inflating a balloon within the trapped area, utilizing the snare technique, deep engagement of a guide catheter, and the child-in-mother technique using the guide extension catheter. The force applied to manually pull back the burr is weaker than when connected to the driveshaft, making percutaneous bailout more difficult if the Rotawire is broken by excessive force. In the present case, attempts were made to retrieve the burr by repeatedly inflating a balloon within the entrapping region from the second guide catheter, but this proved unsuccessful. Moreover, neither deep coronary intubation of the guide catheter nor advancement of a snare to the burr was feasible. Finally, we were able to retrieve the burr by advancing the guide extension catheter until it was in contact with the burr and pulling them together. Therefore, the child-in-mother technique with a guide extension catheter as the initial approach is recommended, as demonstrated in this case.

It is essential to treat proximal calcification when handling calcified lesions in the distal part [7]. Before fracture, the lumen can be widened by ablating calcifications in the proximal RCA using a push-pull technique with an orbital atherectomy system (OAS). If the middle part is then ablated with a Rotablator, it is easier to retrieve the burr even if it fractures. However, it should be noted that even with the use of the OAS, fracture of the OAS crown has been reported [8, 9].

Intravascular lithotripsy can also be used to modulate proximal part calcifications and may allow balloon dilation from a second guiding catheter even after fracture. Even with the emergence of multiple treatment options for calcification, considering the bailout method of fracture remains necessary, as reported.

## 4. Conclusions

In the present report, we present an incident involving burr disconnection from the driveshaft. In clinical practice, being cautious of Rotablator-driven shaft fractures during ablation procedures is crucial.

## Data Availability

Additional data used to support the findings of this study are available from the corresponding author upon request.

## Supporting Information

Supporting Information 1 Video 1: intravascular ultrasound did not pass.

Supporting Information 2 Video 2: angiogram shows separated burr and driveshaft.

Supporting Information 3 Video 3: we observed a separated burr and driveshaft.
